# Supplementary material for: Large-scale filament formation inhibits the activity of CTP synthetase
Source: eLife. 2014 Jul 16;3:e03638. doi: 10.7554/eLife.03638 (PMC4126345; doi:10.7554/eLife.03638)
Supplement: Supplementary file 1. — Model of CtpS polymerization and inhibition. DOI: http://dx.doi.org/10.7554/eLife.03638.034 [file elife03638s001.pdf]

Supplementary Material:
Model of CtpS polymerization and inhibition

Here, we present our mathematical model of CtpS inhibition by CTP-dependent polymerization. We start by describing CtpS polymerization. Then, we model the dependence of polymerization on CTP and UTP concentrations. In particular, we quantify the cooperativity of the CTP-mediated inhibition of CtpS, through the response coefficient of the enzyme to CTP. Finally, we compare our polymerization-based competitive inhibition mechanism to other possible mechanisms of enzyme inhibition. This comparison forms the basis of Fig. 8D in the main text.

1 Polymerization of CtpS

Because our CtpS polymer structure suggests that CtpS forms tetramers before polymerizing, our model assumes that CtpS is pre-organized as tetramers, and focuses on the polymerization of these tetramers. Moreover, the abundance of long polymers observed in our experiments suggests that CtpS polymerization involves a nucleation barrier. Hence, we consider that polymerization of CtpS involves a free-energy cost $f > 0$ per tetramer. This can arise, for example, from a conformational change necessary for polymerization to occur, such as that described in our paper. Besides, we denote by $-E$, with $E > 0$, the polymerization binding free energy. Throughout this analysis, all energies are expressed in units of the thermal energy $k_B T$, where k_B denotes Boltzmann's constant while T denotes absolute temperature. Such models of polymerization with nucleation have been discussed, e.g., in Ref. [1].

First, we show that the fraction ϕ_{np} of CtpS tetramers in the non-polymeric form can be obtained as a function of e^{-f} and of the rescaled total CtpS tetramer concentration $\xi \equiv Kc_t$, where c_t is the total CtpS tetramer concentration in the system, and K is the equilibrium constant of an elementary polymerization step (other than the dimerization step). Then, we derive an estimate of the magnitude of the nucleation barrier f from the observed length of CtpS polymers in cells.

1.1 Fraction of non-polymeric CtpS tetramers

We denote a tetramer of CtpS by A, and a polymer of n tetramers by A_n. Introducing the equilibrium constant K_2 of the first polymerization step, which corresponds to dimerization of two tetramers, and the equilibrium constant K of the successive polymerization steps, we have:

$$2\,A \rightleftharpoons A_2\,, \qquad K_2 = \frac{[A_2]}{[A]^2} = Ce^{E-2f}\,, \tag{1}$$

$$A + A_n \rightleftharpoons A_{n+1}\,, \qquad K = \frac{[A_{n+1}]}{[A_n]\,[A]} = Ce^{E-f}\,, \tag{2}$$

where square brackets denote concentrations. In these equations, C is a constant.

From the definitions of the equilibrium constants K and K_2 in Eqs. 1 and 2, we obtain for all $n \geq 2$

$$[A_n] = K^{n-2} K_2\,[A]^n\,. \tag{3}$$

Thus, the total tetramer concentration c_t in the system, can be expressed as

$$c_t = [A] + \sum_{n=2}^{\infty} n\,[A_n] = [A]\left(1 - e^{-f}\right) + \frac{[A]\;e^{-f}}{(1 - K\,[A])^2}\,, \tag{4}$$

where we have used $K_2/K = e^{-f}$ (see Eqs. 1 and 2), and we have assumed $K[A] < 1$, required for c_t to be finite.

Solving Eq. 4 (which is a third-degree equation in $[A]$) yields the fraction $\phi_{\text{np}} = [A]/c_t$ of non-polymeric CtpS tetramers as a function of e^{-f} and $\xi = Kc_t$. Note that Eq. 4 is easy to solve in the case where $f = 0$, since it then reduces to a second-degree equation in $[A]$.

In the case where $f \gg 1$, the fraction ϕ_{np} (given by the solution of Eq. 4) tends to $\phi_{\text{np}} = 1$ for $\xi < 1$ and $\phi_{\text{np}} = 1/\xi$ for $\xi > 1$. In other words, if the nucleation barrier is large, no polymerization occurs at low c_t and then, with a sharp transition at $\xi = 1$ (i.e., $c_t = 1/K$), polymerization begins. For smaller values of f, the asymptotic behaviors for $\xi \ll 1$ and $\xi \gg 1$ are given, respectively, by $\phi_{\text{np}} = 1$ and $\phi_{\text{np}} = 1/\xi$. The higher f, the steeper the transition between these two asymptotic behaviors. Polymerization starts occurring significantly for $\xi \approx 1$. Examples of plots of ϕ_{np} versus $\xi = Kc_t$, for different values of f, are shown below, in Fig. 8, supplement 4.

An important feature of this polymerization transition is that its onset can become arbitrarily sharp as the nucleation energy is increased. As shown by Fig. 8, supplement 4, a nucleation barrier $f = 9$ (in units of $k_B T$), which corresponds to our estimate of the actual value of f (see below) yields an extremely sharp transition.

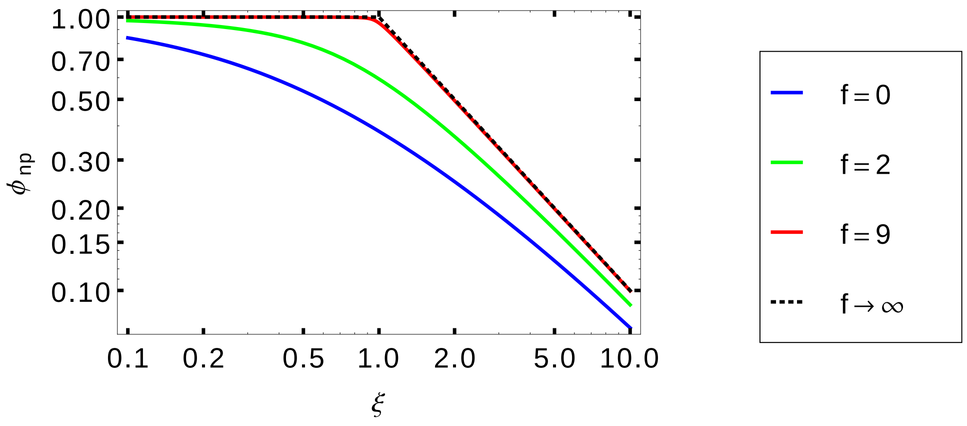

Figure 8, supplement 4: Fraction ϕ_{np} of non-polymeric CtpS tetramers versus $\xi = Kc_t$, where K is the equilibrium constant for polymer growth and c_t is the total tetramer concentration. This fraction ϕ_{np} is plotted for different values of f, in a logarithmic scale.

1.2 Average polymer size

Let us now express the average polymer size from our model. Comparing it to the observed size of CtpS polymers in cells will enable us to estimate the actual value of the nucleation barrier f. We define the average polymer size $\langle n \rangle$, defined per monomer (and often called the weight-average degree of polymerization [1]), as:

$$\langle n \rangle = \frac{\sum_{n=1}^{\infty} n^2 [A_n]}{c_t} \,. \tag{5}$$

This expression can be evaluated using Eqs. 3 and 4, yielding:

$$\langle n \rangle = \frac{(1 - K[A])^3 + e^{-f} K[A] \left(4 - 3K[A] + K^2[A]^2\right)}{\left(1 - e^{-f}\right)(1 - K[A])^3 + e^{-f}\left(1 - K[A]\right)} \,. \tag{6}$$

As explained above, solving Eq. 4 yields the fraction $\phi_{\rm np} = [A]/c_t$ of non-polymeric CtpS tetramers as a function of e^{-f} and $\xi = Kc_t$. Combining this with Eq. 6 yields the average polymer size $\langle n \rangle$ as a function of e^{-f} and $\xi = Kc_t$. We now use this to estimate the value of the nucleation barrier f from the values of the typical polymer length and of ξ in cells.

1.3 Estimate of the nucleation barrier

Ref. [2] shows that the typical length of CtpS polymers in cells is about 400 nm. In addition, the structural study in Fig. 3, supplement 1-C demonstrates that each CtpS tetramer occupies a length 8 nm in the polymer. Hence, there are about 50 tetramers in a typical CtpS polymer *in vivo*.

To estimate f from this average polymer size using our model, we need the value of $\xi = Kc_t$, the renormalized total CtpS tetramer concentration, in cells. The polymerization threshold corresponds to $\xi = 1$ in our model (see above, and Fig. 8, supplement 4). Our experiments indicate that the polymerization threshold of CtpS (measured *in vitro*) is around $1 - 2\ \mu$M (see Fig. 1, supplement 1), while the cellular concentration of CtpS is $2.3\ \mu$M (see Fig. 1, supplement 2). Using these values yields an estimate of ξ in cells between 1.15 and 2.3. (Note that, for this rough estimate, we have ignored the fact that the value of K might be slightly different *in vitro* and *in vivo* because K depends on the UTP and CTP concentrations, as shown in the next section).

Using these estimates for the average polymer length and for the value of ξ in cells, our model yields a nucleation barrier f between 7 and 12 (in units of $k_{\rm B}T$), with a value of 9 for a polymerization threshold of $1.5\ \mu$M. While this estimate of f is quite rough, it argues in favor of a quite large nucleation barrier, which yields sharp polymerization transitions (see Fig. 8, supplement 4). In the following, we will take $f = 9$ when a value of the nucleation barrier is needed for our figures and examples.

2 Influence of UTP and CTP concentrations

2.1 The model

Due to the observed dependence of polymerization on the presence of CTP, we predict that the polymerization binding free energy E increases when CtpS is bound to CTP, which favors polymerization. We assume that each non-polymeric CtpS monomer can bind either to UTP, with dissociation constant $K_{\rm um}$, or to CTP, with dissociation constant $K_{\rm cm}$. Similarly, each CtpS monomer within a polymer can bind to either UTP or CTP, with respective dissociation constants $K_{\rm up}$ and $K_{\rm cp}$.

Given the overlap of the CTP and UTP binding sites, we assume that CTP binding competes with UTP binding. Hence, both polymerized and non-polymeric CtpS monomers exist in three different states: unbound, CTP-bound, and UTP-bound. The (free) energies of the three states of a non-polymeric CtpS monomer are:

1. unbound: $E_1 = E_m$,
2. UTP-bound: $E_2 = E_m - \ln([\text{UTP}]/K_{\rm um})$,
3. CTP-bound: $E_3 = E_m - \ln([\text{CTP}]/K_{\rm cm})$,

where we have introduced the dissociation constants $K_{\rm cm}$ and $K_{\rm um}$ of CTP and UTP, respectively, for non-polymeric CtpS monomers. Therefore, for a non-polymeric monomer, the total free energy F_m, which is defined by $e^{-F_m} = e^{-E_1} + e^{-E_2} + e^{-E_3}$, is:

$$F_m = E_m - \ln\left(1 + \frac{[\text{UTP}]}{K_{\rm um}} + \frac{[\text{CTP}]}{K_{\rm cm}}\right) . \tag{7}$$

Similarly, the free energy F_p of a CtpS monomer within a polymer is

$$F_p = E_p - \ln\left(1 + \frac{[\mathrm{UTP}]}{K_{\mathrm{up}}} + \frac{[\mathrm{CTP}]}{K_{\mathrm{cp}}}\right), \tag{8}$$

where we have introduced the dissociation constants K_{cp} and K_{up} of CTP and UTP, respectively, for polymerized CtpS monomers.

Because our CtpS polymer structure suggests that CtpS forms tetramers before polymerizing, we assume that the polymerization binding free energy E corresponds to $4(F_m - F_p)$, and we have

$$E = E_0 + 4\ln\left(\frac{1 + \frac{[\mathrm{UTP}]}{K_{\mathrm{up}}} + \frac{[\mathrm{CTP}]}{K_{\mathrm{cp}}}}{1 + \frac{[\mathrm{UTP}]}{K_{\mathrm{um}}} + \frac{[\mathrm{CTP}]}{K_{\mathrm{cm}}}}\right), \tag{9}$$

where E_0 is the polymerization binding free energy in the absence of CTP and UTP. Note that we assume that f is not affected by CTP or UTP.

This dependence of E on CTP and UTP concentrations affects CtpS polymerization. Indeed, we saw above that the fraction of non-polymeric CtpS depends on $\xi = Kc_t$, and we have $K \propto e^E$ (see Eq. 2). Given that polymerized CtpS are assumed to be inactive, the CTP and UTP concentrations will in turn affect CtpS activity. Considering that a non-polymerized CtpS monomer is active when it is bound to UTP, the fraction of active CtpS is

$$\phi^* = \phi_{\mathrm{np}} m^* \qquad \text{with} \qquad m^* = \frac{\frac{[\mathrm{UTP}]}{K_{\mathrm{um}}}}{1 + \frac{[\mathrm{UTP}]}{K_{\mathrm{um}}} + \frac{[\mathrm{CTP}]}{K_{\mathrm{cm}}}}. \tag{10}$$

where m^* is the fraction of non-polymeric CtpS monomers that are active. In order to calculate ϕ^* at given UTP and CTP concentrations, we use Eq. 10, and we calculate ϕ_{np} by solving Eq. 4 with the UTP and CTP-concentration dependent binding energy in Eq. 9.

At fixed UTP and CTP concentrations such that $m^* \approx 1$, Eq. 10 shows that $\phi^* \approx \phi_{\mathrm{np}}$. Our discussion of the dependence of ϕ_{np} versus total CtpS concentration in the previous section demonstrates that polymerization ensures negative feedback on CtpS activity from total CtpS levels. Moreover, this negative feedback can be very sensitive, since the onset of decrease in enzyme activity can become arbitrarily sharp as the nucleation energy is increased (see Fig. 8, supplement 4). In particular, this onset is extremely sharp for $f = 9$, which corresponds to our estimate of the actual nucleation barrier in CtpS polymerization (see above). In practice, this negative feedback means that beyond the polymerization threshold, the fraction $\phi^* \approx \phi_{\mathrm{np}}$ of active enzymes will decrease as $1/\xi = 1/(Kc_t)$ when the total concentration of tetramers c_t is increased (see above). In other words, the cellular concentration of active tetramers (which is almost equal to the concentration of non-polymerized tetramers here), given by $\phi^* c_t$, will then saturate and remain constant when c_t is increased beyond the polymerization threshold. These behaviors are illustrated below, in Fig. 8, supplement 5, with $f = 9$, and the sharpness of the onset of the negative feedback is apparent for this value of f.

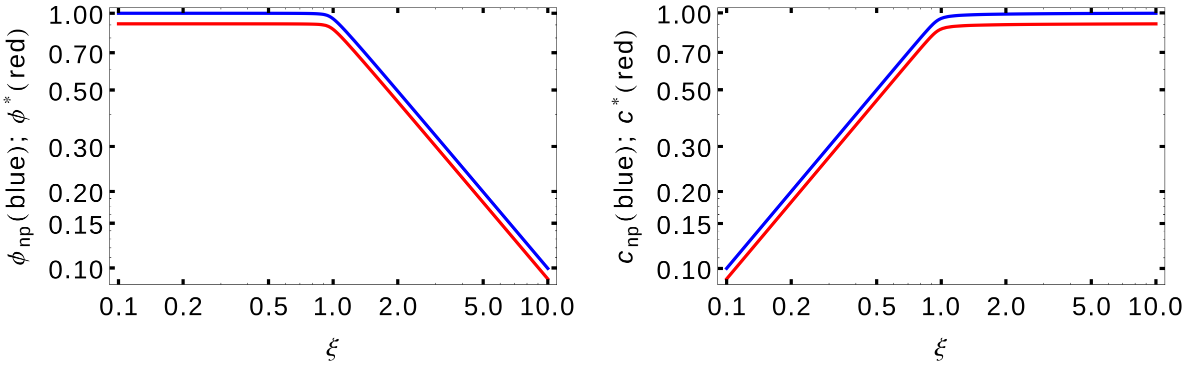

(a) Non-polymeric (ϕ_{np}) and active (ϕ^*) fractions. (b) Non-polymeric (c_{np}) and active (c^*) concentrations.

Figure 8, supplement 5: Plot of the non-polymeric and active fractions (a) and (rescaled) concentrations (b) of CtpS tetramers versus (rescaled) total CtpS tetramer concentration $\xi = Kc_t$, in a logarithmic scale. The parameter values used are the same as in Fig. 8D in the main text (see below for details), and the concentration of CTP is zero.

2.2 Polymerization threshold as a function of CTP concentration at high UTP concentration

As shown in Section 1, the onset of polymerization occurs at $\xi \approx 1$. To model polymerization as a function of CTP concentration, we expressed this condition explicitly in terms of CTP concentration. Given that $\xi = Kc_t = Ce^{E-f}c_t$ (see Eq. 2), we obtain, using Eq. 9,

$$\xi = \xi_0 \left(\frac{1 + \frac{[\mathrm{UTP}]}{K_{\mathrm{up}}} + \frac{[\mathrm{CTP}]}{K_{\mathrm{cp}}}}{1 + \frac{[\mathrm{UTP}]}{K_{\mathrm{um}}} + \frac{[\mathrm{CTP}]}{K_{\mathrm{cm}}}} \right)^4 , \tag{11}$$

where

$$\xi_0 = K_0 c_t = Ce^{E_0 - f} c_t . \tag{12}$$

ξ_0 is the value of ξ in the absence of CTP and UTP, all other conditions being the same.

Next, we determined the polymerization threshold, taken to be $\xi = 1$, as a function of CTP concentration at high UTP concentration. We expect that UTP binds strongly to non-polymeric CtpS, but only very weakly to polymerized CtpS, such that $K_{\mathrm{um}} \ll K_{\mathrm{up}}$. By contrast, we assume that CTP binds weakly to non-polymeric CtpS, and strongly to polymerized CtpS, such that $K_{\mathrm{cm}} \gg K_{\mathrm{cp}}$. We choose a UTP concentration high enough for the fraction m^* of non-polymeric monomers that are active to be close to one, but still low enough for UTP binding to the polymer to be weak. In other words, we assume that

$$K_{\mathrm{um}} \ll [\mathrm{UTP}] \ll K_{\mathrm{up}} \tag{13}$$

and that

$$\frac{[\mathrm{UTP}]}{K_{\mathrm{um}}} \gg \frac{[\mathrm{CTP}]}{K_{\mathrm{cm}}} . \tag{14}$$

The latter condition is likely true over a large range of CTP concentrations since we expect non-polymeric CtpS to have a higher affinity for UTP than for CTP, i.e., $K_{\mathrm{um}} < K_{\mathrm{cm}}$.

In this case, m^* is close to 1 and we have

$$\xi \approx \xi_0 \left(\frac{K_{\mathrm{um}}}{[\mathrm{UTP}]} \right)^4 \left(1 + \frac{[\mathrm{CTP}]}{K_{\mathrm{cp}}} \right)^4 . \tag{15}$$

In addition, we assume that

$$\xi_0 \left(\frac{K_{\mathrm{um}}}{[\mathrm{UTP}]}\right)^4 \ll 1\,, \tag{16}$$

so that polymerization does not occur in the absence of CTP and will be induced by increasing the CTP concentration. In this case, we expect to see strong regulation of CtpS activity by polymerization.

When CtpS is more active than the metabolic needs of the cell require, CTP will accumulate, so we determined the effect of an increase in CTP concentration on CtpS activity, while remaining in the validity domain of Eq. 14. For ξ to reach 1 (and thus for polymerization to start), it is necessary to increase CTP concentration so that $[\mathrm{CTP}] \gg K_{\mathrm{cp}}$. Hence, in the range of CTP concentrations where polymerization occurs, Eq. 15 simply becomes:

$$\xi \approx \xi_0 \left(\frac{K_{\mathrm{um}}}{K_{\mathrm{cp}}[\mathrm{UTP}]}\right)^4 [\mathrm{CTP}]^4\,. \tag{17}$$

This simple form of CTP-dependence of ξ allows for a straightforward analysis of the cooperativity of the inhibition of CtpS by CTP, which is presented below.

2.3 Response coefficient

In order to quantify the cooperativity of CtpS inhibition by CTP-dependent polymerization, we calculate the response coefficient for inhibition of CtpS by CTP. We define this response coefficient as the ratio of CTP concentration yielding 10% active enzymes to that yielding 90% active enzymes. We consider active enzymes to be the non-polymeric and UTP-bound ones. Standard noncooperative inhibition, for which the fraction of active enzymes is $\phi^* = 1/(1 + [\mathrm{CTP}]/K_{\mathrm{CTP}})$, yields a response coefficient of 81, and smaller values indicate cooperative inhibition. In the particular case of a Hill function, our response coefficient r can be related to the Hill coefficient n through $n = \ln(81)/\ln(r)$.

As discussed, for $f \gg 1$, polymerization begins abruptly when $\xi = Kc_t = 1$, below which $\phi_{\mathrm{np}} = 1$ and above which $\phi_{\mathrm{np}} = 1/\xi$. Thus, Eq. 17 entails that, if $f \gg 1$, there is an abrupt transition from $\phi_{\mathrm{np}} = 1$ to $\phi_{\mathrm{np}} \propto [\mathrm{CTP}]^{-4}$. Hence, since $m^* = 1$ in the regime of [UTP] we consider, there is also an abrupt transition from $\phi^* = 1$ to $\phi^* \propto [\mathrm{CTP}]^{-4}$. If f is smaller, one still has a transition between these asymptotic regimes, but it is less abrupt. Note that the fourth-power dependence of ϕ^* on $[\mathrm{CTP}]^{-1}$ comes directly from the fact that CtpS polymerizes in its tetrameric form.

As a consequence, the minimum response coefficient for ϕ_{np} (or ϕ^*) as a function of CTP concentration corresponds to a sharp transition from $\phi_{\mathrm{np}} = 1$ to $\phi_{\mathrm{np}} \propto [\mathrm{CTP}]^{-4}$. The minimum response coefficient is therefore $\sqrt{3} \approx 1.7$, corresponding to an effective Hill coefficient of 8: this value indicates a very high cooperativity. The response coefficient tends toward this minimum value for $f \gg 1$, provided the assumptions in Sec. 2.2 are satisfied.

If f is smaller (but all the assumptions in Sec. 2.2 remain true), the response coefficient will become larger as the transition from $\phi_{\mathrm{np}} = 1$ to $\phi_{\mathrm{np}} \propto [\mathrm{CTP}]^{-4}$ becomes smoother. In particular, for $f = 0$, the response coefficient is 3.3, corresponding to an effective Hill coefficient of about 3.7. However, the response coefficient can be larger than 3.3 if some of the assumptions of Sec. 2.2 are violated. For instance, the response coefficient can become much larger (less cooperative) if Eq. 16 is not satisfied, as polymerization then occurs in the absence of CTP and is no longer triggered by increasing CTP concentration.

While response coefficients and Hill coefficients are useful to quantify the sharpness of enzyme regulation, it is important to note that within our novel polymerization-based negative feedback mechanism, enzyme activity is not a simple Hill function of CTP concentration. In particular, the onset of the decrease of activity can become arbitrarily sharp as the nucleation barrier is increased,

since this decrease is driven by the onset of polymerization. For instance, a more local response coefficient, calculated between 50% and 90% enzyme activities (instead of 10% and 90%), would be as low as about 1.2 for $f \gg 1$, corresponding to an effective Hill coefficient of about 15. This steep onset of enzyme inhibition is fundamentally different from the case of enzyme oligomers that cooperatively bind an inhibitor, which would yield a simple Hill function.

3 Comparison of different modes of enzyme inhibition

CTP-dependent CtpS polymerization gives rise to highly cooperative competitive inhibition. We compared this mechanism (denoted by C-P) to other possible types of enzyme inhibition:
- non-competitive inhibition with CTP-dependent polymerization (NC-P)
- competitive inhibition without polymerization (C-NP)
- non-competitive inhibition without polymerization (NC-NP) .

3.1 Non-competitive inhibition with CTP-dependent polymerization (NC-P)

In the case of non-competitive inhibition with CTP-dependent polymerization, we can simply adapt the model developed above for the case of competitive inhibition with CTP-dependent polymerization. The only change is that both non-polymeric monomers and monomers in polymers can now be present in 4 states instead of 3, the new state being "bound to both UTP and CTP". CtpS is assumed to be inactive in this state. We assume for simplicity that the binding affinity of UTP for a CTP-bound enzyme is the same as for an unbound enzyme, and similarly that the binding affinity of CTP for a UTP-bound enzyme is the same as for an unbound enzyme, albeit these binding affinities still depend on polymerization state. Hence, we have

$$\xi = \xi_0 \left[\frac{\left(1 + \frac{[\mathrm{UTP}]}{K_{\mathrm{up}}}\right)\left(1 + \frac{[\mathrm{CTP}]}{K_{\mathrm{cp}}}\right)}{\left(1 + \frac{[\mathrm{UTP}]}{K_{\mathrm{um}}}\right)\left(1 + \frac{[\mathrm{CTP}]}{K_{\mathrm{cm}}}\right)} \right]^4 , \tag{18}$$

instead of Eq. 11, with a polymerization threshold that still occurs at $\xi = 1$. In addition, assuming that non-polymeric CtpS is inactive when bound both to CTP and UTP, we have

$$\phi^* = \phi_{\mathrm{np}} m^* \qquad \text{with} \qquad m^* = \frac{\frac{[\mathrm{UTP}]}{K_{\mathrm{um}}}}{\left(1 + \frac{[\mathrm{UTP}]}{K_{\mathrm{um}}}\right)\left(1 + \frac{[\mathrm{CTP}]}{K_{\mathrm{cm}}}\right)} , \tag{19}$$

instead of Eq. 10.

As above, we assume conditions such that the fraction m^* of non-polymeric monomers that are active is close to one: here, it means $[\mathrm{UTP}] \gg K_{\mathrm{um}}$ and $[\mathrm{CTP}] \ll K_{\mathrm{cm}}$. As above also, we choose the UTP concentration such that UTP binding to the polymer remains weak: $K_{\mathrm{up}} \gg [\mathrm{UTP}]$. Hence, as in the case of competitive inhibition, we choose to work in the UTP regime $K_{\mathrm{um}} \ll [\mathrm{UTP}] \ll K_{\mathrm{up}}$.

It is important to note that the condition on CTP concentration that is necessary for m^* to be close to one (namely, $[\mathrm{CTP}] \ll K_{\mathrm{cm}}$) is different from the condition that was necessary in the case of competitive inhibition (Eq. 14). Given that we are in the regime where $[\mathrm{UTP}] \gg K_{\mathrm{um}}$, the condition $[\mathrm{CTP}] \ll K_{\mathrm{cm}}$ is *more restrictive* than Eq. 14. Qualitatively, the fact that here CTP can bind to a UTP-bound enzyme and inactivate it provides an additional mechanism for CTP to decrease enzyme activity. This can reduce the CTP concentration range over which non-polymeric enzymes remain active.

If all these conditions are met at the onset of polymerization, and if, in addition, Eq. 16 is also satisfied, we find exactly the same results as in the above-studied competitive inhibition case (in

particular, Eq. 17 holds). Hence, in this case, all the above results (in particular the minimum response coefficient equal to $\sqrt{3}$) hold, and the competitive and non-competitive inhibitions yield the same behaviors.

However, if the condition $[\mathrm{CTP}] \ll K_{\mathrm{cm}}$ is not met at the onset of polymerization, while the other conditions and Eq. 14 are satisfied, the competitive and non-competitive cases will be different. Using only the condition $K_{\mathrm{um}} \ll [\mathrm{UTP}] \ll K_{\mathrm{up}}$, Eq. 18 becomes

$$\xi = \xi_0 \left(\frac{K_{\mathrm{um}}}{[\mathrm{UTP}]} \right)^4 \left(\frac{1 + \frac{[\mathrm{CTP}]}{K_{\mathrm{cp}}}}{1 + \frac{[\mathrm{CTP}]}{K_{\mathrm{cm}}}} \right)^4 , \tag{20}$$

which grows with increasing CTP concentrations, saturating at

$$\xi = \xi_0 \left(\frac{K_{\mathrm{um}}}{[\mathrm{UTP}]} \right)^4 \left(\frac{K_{\mathrm{cm}}}{K_{\mathrm{cp}}} \right)^4 . \tag{21}$$

In particular, if this value is below 1, polymerization will never occur: then, the behavior will be dramatically different from that in the competitive case and all cooperativity will disappear.

Therefore, depending on parameters and on the UTP concentration, the two types of inhibition, competitive and non-competitive, with CTP-dependent polymerization, can yield either similar or very different results. Specifically, the range of UTP and CTP concentrations where high cooperativity is expected is reduced in the case of non-competitive inhibition. High cooperativity is therefore more likely in the case of competitive inhibition, and should be more robust.

3.2 Competitive inhibition without polymerization (C-NP)

In the case of competitive inhibition without polymerization, we simply have the active CtpS fraction:

$$\phi^* = \frac{\frac{[\mathrm{UTP}]}{K_{\mathrm{um}}}}{1 + \frac{[\mathrm{UTP}]}{K_{\mathrm{um}}} + \frac{[\mathrm{CTP}]}{K_{\mathrm{cm}}}} . \tag{22}$$

This corresponds to Eq. 10 with $\phi_{\mathrm{np}} = 1$ since here we only have non-polymeric enzymes.

Here, when increasing CTP concentration for $[\mathrm{UTP}] \gg K_{\mathrm{um}}$, we find that $[\mathrm{CTP}] \gg K_{\mathrm{cm}}$ is necessary for ϕ^* to decrease below one. Hence, in the transition region, we have

$$\phi^* \approx \frac{1}{1 + \frac{[\mathrm{CTP}] K_{\mathrm{um}}}{[\mathrm{UTP}] K_{\mathrm{cm}}}} . \tag{23}$$

This implies that ϕ^* will start decreasing at $[\mathrm{CTP}] \approx [\mathrm{UTP}] K_{\mathrm{cm}} / K_{\mathrm{um}}$, and also that the response coefficient will be very close to the noncooperative value of 81 (indeed, Eq. 23 is exactly a standard Hill curve with response coefficient 81, or Hill coefficient 1).

3.3 Non-competitive inhibition without polymerization (NC-NP)

Finally, in the case of non-competitive inhibition without polymerization, we have:

$$\phi^* = \frac{\frac{[\mathrm{UTP}]}{K_{\mathrm{um}}}}{\left(1 + \frac{[\mathrm{UTP}]}{K_{\mathrm{um}}}\right) \left(1 + \frac{[\mathrm{CTP}]}{K_{\mathrm{cm}}}\right)} . \tag{24}$$

This corresponds to Eq. 19 with $\phi_{\mathrm{np}} = 1$ since here we only have non-polymeric enzymes.

Here, for $[\mathrm{UTP}] \gg K_{\mathrm{um}}$, we have

$$\phi^* \approx \frac{1}{1+\frac{[\mathrm{CTP}]}{K_{\mathrm{cm}}}}\,. \tag{25}$$

This implies that ϕ^* will start decreasing at $[\mathrm{CTP}] \approx K_{\mathrm{cm}}$, and here too the response coefficient will be very close to the noncooperative value of 81 (indeed, Eq. 25 is exactly a standard Hill curve with response coefficient 81, or Hill coefficient 1).

4 Parameter values chosen in Fig. 8D

Fig. 8D in the main text compares the different mechanisms of enzyme inhibition described above. It presents the fraction ϕ^* of active (i.e., nonpolymerized and UTP-bound) CtpS, plotted versus CTP concentration on a logarithmic scale.

In all cases, we chose a fixed UTP concentration equal to K_{cp}, and we took $K_{\mathrm{up}} = 10\,K_{\mathrm{cp}}$, $K_{\mathrm{um}} = 0.1\,K_{\mathrm{cp}}$, $K_{\mathrm{cm}} = 100\,K_{\mathrm{cp}}$. In addition, we took the value estimated above for the free-energy cost for the polymer configuration of each CtpS tetramer: $f = 9$ (in units of $k_{\mathrm{B}}T$). Besides, we chose $\xi_0 = 1$, which means that the total concentration of CtpS tetramers is equal to $1/K_0$ (cf. Eq. 12), where K_0 is the equilibrium constant of the addition of one extra CtpS tetramer to a polymer in the absence of UTP and CTP.

The values of these parameters were chosen such that the conditions discussed in Sec. 2.2 (in the competitive polymerizing, C-P, case) are satisfied. First, Eq. 13 and Eq. 16 are satisfied. Furthermore, the condition Eq. 14 is valid for $[\mathrm{CTP}] \ll 1000\,K_{\mathrm{cp}}$. This condition is valid in a wide range of CTP concentrations where polymerization occurs, since the polymerization threshold, obtained by setting $\xi = 1$ in Eq. 17, is $[\mathrm{CTP}] \approx 11\,K_{\mathrm{cp}}$ here. It can be seen on the C-P curve of Fig. 8D that the onset of polymerization is at $[\mathrm{CTP}] \approx 11\,K_{\mathrm{cp}}$, and that a transition to a less steep decrease of ϕ_{np} occurs at $[\mathrm{CTP}] \approx 1000\,K_{\mathrm{cp}}$, when the active fraction of non-polymeric tetramers starts to significantly decrease.

The response coefficients r, in the cases involving polymerization, are $r_{\text{C-P}} = 1.8$ for competitive inhibition (the main case studied here) and $r_{\text{NC-P}} = 2.0$ for noncompetitive inhibition (the case studied in Sec. 3.1): both demonstrate high cooperativity, close to the maximum cooperativity discussed above (for which $r = \sqrt{3} \approx 1.7$). Conversely, the response coefficients of both non-polymerizing cases are equal to 81, indicating absence of cooperativity.

References

[1] D. H. Zhao and J. S. Moore. Nucleation-elongation: a mechanism for cooperative supramolecular polymerization. *Org. Biomol. Chem.*, 1(20):3471–3491, 2003.

[2] M. Ingerson-Mahar, A. Briegel, J. N. Werner, G. J. Jensen, and Z. Gitai. The metabolic enzyme CTP synthase forms cytoskeletal filaments. *Nat. Cell Biol.*, 12(8):739–746, 2010.
